# Severe Immune Thrombocytopenia Following MMR Vaccination with Rapid Recovery: A Case Report and Review of Literature

**DOI:** 10.2147/IMCRJ.S286335

**Published:** 2020-12-15

**Authors:** Majd Akbik, Dima Naddeh, Anas A Ashour, Azzam Ashour

**Affiliations:** 1 Al-Thumama Health Center, Primary Health Care Corporation, Doha 26555, Qatar; 2 Muaither Health Center, Primary Health Care Corporation, Doha 26555, Qatar; 3 College of Medicine, QU Health, Qatar University, Doha 2713, Qatar; 4 Medical Education, Hamad Medical Corporation, Doha 3050, Qatar

**Keywords:** immune thrombocytopenia, MMR vaccine, thrombocytopenia, case report

## Abstract

Immune thrombocytopenia (ITP) is an autoimmune disease that occurs following viral illnesses and may also infrequently occur after measles, mumps, and rubella (MMR) vaccination. ITP typically presents with the sudden appearance of a petechial rash, bruising, and/or bleeding in an otherwise healthy-appearing child. However, ITP following MMR vaccine does not commonly cause severely depleted platelets. We report a case of ITP after MMR vaccination in a 13-month-old baby boy, who presented with petechial rash all over his body. The child had severe thrombocytopenia but was successfully treated with a single dose of intravenous immunoglobulin without complications. The study highlights that ITP post-MMR vaccine is an easily treatable condition.

## Introduction

Immune thrombocytopenia (ITP) is defined by thrombocytopenia that is not associated with other hematologic abnormalities. While it commonly results in severe thrombocytopenia, it is usually not linked to life-threatening bleeding in children.^1^ ITP can be caused by systemic diseases, immune deficiencies or viruses.^1^ Although rare, vaccines such as measles, mumps, and rubella (MMR) vaccination is a recognized cause of ITP.^2^ ITP post-MMR incidence is reported as 2.5 cases per 100,000 vaccinations.^2^ ITP caused by MMR vaccine usually develops 2 to 6 weeks following the vaccine but is usually self-limiting with mild to moderate thrombocytopenia.^3^ Complications such as severe hemorrhage or very low platelets are treated with Intravenous Immunoglobulins (IVIG) or glucocorticoids.^1^ Another alternative first-line therapy that could be used in selected patients is Anti-D immunoglobulin (Anti-D). However, Anti-D has no effect and should not be used in children who have Rhesus factor negative blood or in children who underwent splenectomy.^4^ In children with chronic ITP who continue to have thrombocytopenia for more than 12 months, other second-line treatments such as rituximab, thrombopoietin receptor agonist, and splenectomy might be indicated.^5^ Herein, we report a case of ITP caused by MMR vaccine presenting as severe thrombocytopenia which was treated successfully with IVIG.

## Case Presentation

A 13-month-old baby boy, full-term, normal vaginal delivery with no neonatal intensive care unit admission, presented with rash for one day. The baby was doing well until the mother noticed rash on his feet that spread to the back, abdomen, neck and face. The rash was pinpoint, purple in colour, non-itchy and not preceded by trauma. There was no bleeding from the mouth or nose, no change in urine colour, and no blood in stool. The mother did not report any fever, hypoactivity, poor feeding, diarrhea, or vomiting. The patient had no history of recent upper respiratory tract infection or any viral infection.

Past medical history was unremarkable with no recent medications used. The boy received his one-year MMR vaccine 3 weeks before the presentation. The patient lives with the father and the mother with no history of substance abuse in household or signs of Injuries, abuse or neglect. Family history is negative for bleeding disorders.

On examination, the patient had a petechial rash and bruising on the head, back, upper and lower limbs, with some on the abdomen as well. There was no palpable lymph node or hepatosplenomegaly. Examination otherwise was normal.

Complete blood count (CBC) showed white blood cells of 9.3 ×10^9^/L (reference range: 4.0–12.0×10^9^/L), hemoglobin of 12.8 g/L, and mean corpuscular volume of 71.6 fL. Platelets was 1000/μL (reference range 150 000–400 000/μL). Peripheral smear showed red blood cells with mild microcytosis and anisocytosis, some reactive lymphocytes with few atypical forms, and severe thrombocytopenia with few large reactive platelets consistent with ITP or infection.

Other laboratory investigations included C reactive protein (<0.3), Bilirubin (12 mmol/L), and albumin (47 mmol/L). Urine dipstick was negative. Blood virology was negative for Epstein-Barr virus with positive Cytomegalovirus IgG.

The patient was diagnosed with Post-MMR ITP after excluding other common causes. Due to the very low platelet count and the risk of bleeding, the patient was admitted in general pediatrics ward of Hamad General Hospital for monitoring and was given IVIG 1 g/kg over 12 hours (one dose). After 24 hours of treatment, platelet count increased to 59,000/μL. The patient was discharged from the hospital and the family were advised to avoid trauma as the patient is still considered a high risk for bleeding. The patient was seen in hematology clinic for follow-up after one week. He was doing well, and the CBC showed platelets of 495,000/μL.

## Discussion

Although ITP post-MMR vaccine is previously reported in the literature, it usually presents with mild to moderate thrombocytopenia.^3^ In our case, the patient had severe thrombocytopenia with a platelet count reaching a nadir of 1000/μL. More importantly, it also highlights that even with very low platelet counts, ITP following vaccination is a treatable condition with a low risk of complications. This was evident in the rapid rise of platelet count after treatment and the absence of major or life-threatening bleeding. The benign course of the condition is consistent with previously published reports.^3^ In the study published by Miller et al, ITP following MMR vaccine showed to have a more self-limited course and a shorter duration of hospitalization (3 vs 5 days) compared to non-vaccine-related ITP.^6^ Moreover, the incidence of post-MMR vaccine ITP is reported in the literature as 2.5 cases per 100,000 vaccinations which is a much lower rate when compared to ITP following rubella virus infection (6/100 000) or measles infection (33/100 000).^2^,^7^,^8^ Few case reports have described developing ITP following other vaccinations such as tetanus-diphtheria-acellular pertussis, varicella, and hepatitis A. However, a study by O’Leary et al reported no increased risk of ITP following these vaccinations compared to the general population.^9^ Consequently, A causal relationship can only be identified following MMR vaccination. While physicians should be aware of the association between the MMR vaccine and ITP, the milder course and the rare incidence should encourage physicians not to withhold the vaccination. Nevertheless, patients’ families should be educated about the possible adverse effects of vaccination.

The pathogenesis of Post-vaccination ITP is similar to drug-induced ITP in which Antibodies that are formed following vaccination cross-react with platelets antigens such as GP Ib/IX, GP Ia/IIa, and GP VI.^10^ This mechanism can be confirmed by the fact that 79% of patients with ITP following vaccination have antibody-coated platelets.^11^ MMR vaccine is believed to cause ITP by the same mechanism. A study published by Okazaki et al demonstrated an evidence of the presence of anti-measles and anti-rubella virus antibodies attached to the platelets of a child who suffered from post-MMR ITP.^12^ Furthermore, it is believed that the immaturity of the immune network in younger ages is the reason behind the higher incidence of post-vaccination ITP when compared to teenagers and adults.^13^ As the vaccine could theoretically trigger ITP when obtaining booster doses, an essential part of the long-term management of such cases is the decision whether to administer the second dose of MMR or not. A systematic review of published studies reported that out of 37 children received MMR vaccine following vaccine-related ITP, none developed a recurrence of ITP following the second dose of MMR. Also, in 94 children with non-vaccine related ITP, none developed the condition following the first dose of the MMR vaccine.^3^ Based on these findings, The American Society of Hematology recommends vaccinating children with previous history of ITP unless the child received the first dose of MMR and has a serologic evidence of immunity.^14^ This recommendation is in view of the fact that the benefits of the vaccine greatly outweighs the risks of developing ITP.

## Conclusion

Even though it is important to highlight that ITP can be caused by MMR vaccination, the incidence of ITP following vaccination is significantly lower than the incidence of the condition following the infections prevented by this vaccine. Consequently, post-MMR vaccine ITP should not limit the universal MMR vaccination of children.
